# Combined use of SpineJack and microwave ablation with CT and C-arm in the treatment of vertebral fractures in oncologic patients: a case- based technical note

**DOI:** 10.1093/omcr/omaf019

**Published:** 2025-04-28

**Authors:** Claudio Pusceddu, Isabel Morera Fuster, Jesús Ares-Vidal, Jesús Lafuente Baraza, David Rodríguez Rubio, José María Maiques Llácer, Eliodoro Faiella, Claudio Cau, Pierluigi Rinaldi, Albert Solano López, Salvatore Marsico

**Affiliations:** Department of Radiology, Mater Olbia Hospital, SS 125 Orientale Sarda, 07026 Olbia SS, Italia; Department of Radiology, Hospital del Mar, Passeig Marítim, 25-29 Barcelona, 08003, Spain; Department of Radiology, Hospital del Mar, Passeig Marítim, 25-29 Barcelona, 08003, Spain; Functional Spine Unit - Neurosurgery Department - Hospital del Mar - Passeig Marítim, 25-29 Barcelona, 08003, Spain; Functional Spine Unit - Neurosurgery Department - Hospital del Mar - Passeig Marítim, 25-29 Barcelona, 08003, Spain; Department of Radiology, Hospital del Mar, Passeig Marítim, 25-29 Barcelona, 08003, Spain; Department of Radiology and Interventional Radiology, Fondazione Policlinico Universitario Campus Bio-Medico, Via Alvaro del Portillo, 00128 Rome, Italy; Department of Radiology, Mater Olbia Hospital, SS 125 Orientale Sarda, 07026 Olbia SS, Italia; Department of Radiology, Mater Olbia Hospital, SS 125 Orientale Sarda, 07026 Olbia SS, Italia; Department of Radiology, Hospital del Mar, Passeig Marítim, 25-29 Barcelona, 08003, Spain; Department of Radiology, Hospital del Mar, Passeig Marítim, 25-29 Barcelona, 08003, Spain

**Keywords:** microwave ablation, SpineJack, bone metastasis, vertebral stabilization, CT imaging, C-arm fluoroscopy, lung cancer

## Abstract

This technical report highlights the combined treatment of bone metastasis using CT imaging and C-arm fluoroscopy to guide microwave thermal ablation and the SpineJack system. The integration of these imaging techniques was crucial for achieving local tumor control and restoring vertebral stability in cases of pathological fractures associated with metastatic disease. CT imaging ensured accurate tumor volume measurement and ablation, safe needle placement, and secure positioning of protective devices, while C-arm fluoroscopy provided real-time guidance for the correct positioning of the SpineJack implants, monitoring their expansion, and ensuring controlled cement application. Although the combination of these techniques has been increasingly utilized, this is the first detailed report to focus on their combined use in treating pathological fractures within a metastatic setting.

## Introduction

Bone metastases in cancer patients often cause pain, instability, and neurological deficits, greatly affecting quality of life. Pathological fractures, particularly in the spine, increase the risk of vertebral collapse and related morbidity. Standard treatments like chemotherapy and radiotherapy may not always provide sufficient local control or spine stabilization, especially in advanced cases [1, 2].

Minimally invasive techniques, such as microwave ablation, offer effective local tumor control, while vertebral cementation methods like vertebroplasty, kyphoplasty, and implants provide spinal stabilization and pain relief [3–6].

This report presents the case of a 52-year-old male with lung cancer and a pathological fracture of the T8 vertebra due to metastasis.

The patient was treated with microwave ablation and the SpineJack system. The treatment was chosen due to an isolated, irregular compression fracture, with the patient’s age and spinal condition supporting vertebral reconstruction with the SpineJack system. Tumor debulking was first performed using microwave ablation for local control.

Computed Tomography (CT) imaging was crucial in measuring the tumor volume and guiding the placement of ablation needles. C-arm fluoroscopy enabled accurate placement, real-time expansion of the SpineJack implants, and controlled cement application. This report provides insights into the combination of these advanced techniques for managing complex spinal metastases in cancer patients.

## Case presentation and technical note

**Figure 1:**
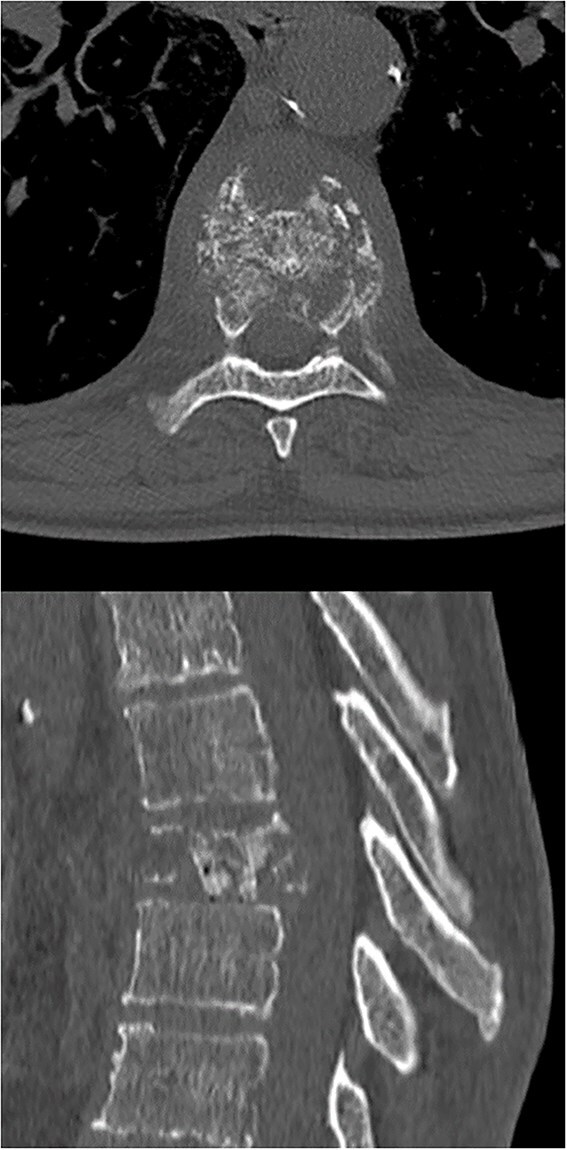
A computed tomography scan with axial (upper image) and sagittal plane (lower image) reconstructions with ‘bone’ filter revealing a compression fracture of the T8 vertebral body. The scan showed multiple fracture lines with a mixed lytic-sclerotic tumor component, with the lytic aspect being more prominent in the anterior portion of the vertebral body. There was also mild posterior wall prominence, but without significant stenosis of the vertebral canal.

In this report, we present the case of a 52-year-old male patient with primary lung adenocarcinoma diagnosed in 2023 who arrived at our hospital with severe, debilitating shoulder pain (VAS 9) in the low dorsal area. A CT scan was performed, revealing a compression fracture of the T8 vertebral body, which had not been evident in previous assessments (Fig. 1). The scan showed multiple fracture lines with a mixed lytic-sclerotic tumor component, with the lytic aspect being more prominent in the anterior portion of the vertebral body. There was also mild posterior wall prominence, but without significant stenosis of the vertebral canal.

**Figure 2:**
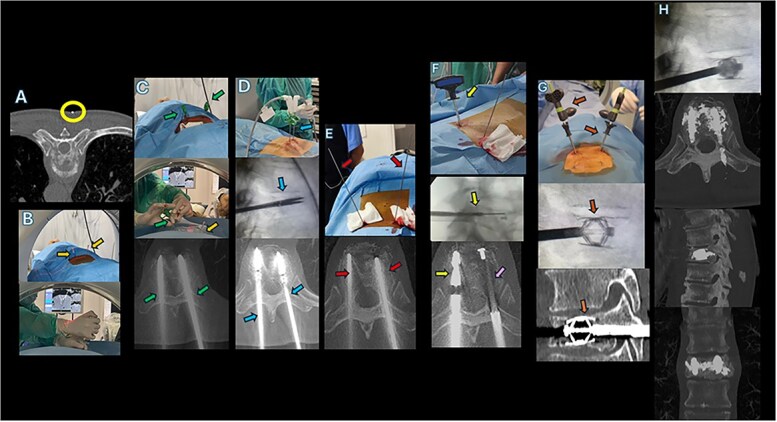
2A: Marker placement. Yellow circle: Metallic markers are inserted into the vertebra at predetermined positions after measuring from the midline to the lateral edge of the vertebra, verified by an axial CT scan. 1B: Needle placement and Anesthesia Orange arrows: Local anesthesia in periosteum (bupivacaine). 2C: Needle insertion for Vertebroplasty:Green arrows: 11-gauge vertebroplasty needles are inserted transpedicular, guided under real-time fluoroscopy to reach the anterior third of the vertebral body with final CT scan check. 2D: Microwave ablation (MWA): Blue arrows: A 20 cm microwave antenna is inserted through the vertebroplasty needle, ensuring the antenna covers the entire lesion. Ablation is performed using a 2.45 GHz microwave generator to target the tumor. CT scan is crucial to obtain a correct measurement of ablation volume to avoid complications. 2E: Kirschner wire insertion red arrows: Kirschner wire is inserted into the vertebral body after removing the vertebroplasty needle’s inner cannula, advancing under fluoroscopic guidance to the anterior third of the vertebral body. CT scan is crucial to rule out any misalignment or to assess if and how the creation of the subsequent channels can be improved. 2F: Drilling and Bone Canal preparation: Yellow arrow: A drill is advanced under fluoroscopic guidance along the Kirschner wire. Pink arrow: An acrylic plug is left in place after the drill is removed, allowing preparation for the subsequent implant channel preparation. 2G: SpineJack® implantation:Brown arrows: Two SpineJack® expandable titanium implants are inserted and gradually deployed under fluoroscopic guidance, restoring vertebral height and reducing kyphosis. 2H: Post-procedural fluoroscopy image and multiplanar CT scan: Post cementation lateral fluoroscopic imaging and CT scan, with multiplanar (MPR) reconstruction scan to assess implant placement, cement distribution, and symmetry of vertebral body expansion. This scan also helps identify potential complications such as cement leakage.

It was decided to perform a bilateral vertebral microwave ablation and SpineJack implantation at the same level to enable debulking of the lesion, and vertebral stabilization in a single procedure (Fig. 2).

**1. Antibiotic Prophylaxis** 

Administer cefazolin 2 g intravenously (IV) 30 minutes before the procedure to prevent infection.

**2. Pre-procedural Imaging and Reper Placement** 

● Positioning of the C-arm and CT: A portable surgical C-arm is positioned laterally, adjacent to the CT table. Serial CT scans are performed, with real-time evaluation using the C-arm.

● Initial CT scan: Perform a CT scan to assess the lesion’s size, location, and radiological characteristics. This helps in procedural planning.

● Measurement: Measure from the midline to the lateral edge of the vertebra to determine the optimal placement for metallic markers.

● Marker Placement: Insert metallic markers into the vertebra at predetermined positions (yellow circle in Fig. 2A).

● CT Confirmation: Verify marker placement with a repeat CT scan. If satisfactory, mark the skin surface using a sterile marker to guide needle insertion.

**3. Anesthesia** 

● Sedation: The patient is positioned prone, and conscious sedation is administered using continuous intravenous infusion of fentanyl citrate (0.1 mg/2 mL diluted 1:10 with saline).

● Local Anesthesia:

○ Lidocaine (1–2%) is administered subcutaneously at the incision site using a 22-gauge intramuscular needle.

○ Using an 18-gauge spinal needle (88 mm), infiltrate bupivacaine (0.25%–0.5%) around the periosteum to provide deeper anesthesia. Needle placement is verified with fluoroscopic and CT guidance (Orange arrows in Figs. 2B,C).

**4. Needle Selection and Insertion** 

a. Vertebroplasty Needle Preparation

● Spinal Needle: CT guided transpedicular insertion 11-gauge (G) vertebroplasty needles to introduce cement into the vertebral body. These needles ensure precise and controlled cement delivery.

● Incisions: Make small skin incisions using a scalpel perpendicular to the spinal needles for easier penetration.

b. Insertion of Needles

● Advance the 11G vertebroplasty needles, by real time fluoroscopic guidance, through the incision, guiding them under fluoroscopy to the anterior third of the vertebral body (Green arrows Fig. 2C).

● Needle Orientation: Ensure the arrow on the vertebroplasty needle points medially for optimal positioning.

**5. Microwave Ablation (MWA) (**  **Fig. 1D****)** 

● Microwave Antenna Selection: Insert a 20 cm microwave antenna (blue arrows in Fig. 1D) coaxially through the vertebroplasty needle. The antenna typically has a 14-gauge (G) diameter and features a mini choked water-cooling system to prevent overheating.

● Before ablation, perform a CT scan to ensure the correct positioning of the needles and calculate the precise volume of ablation required. This step is crucial to achieve optimal ablation and avoid complications.

● Ablation: Advance the microwave antenna as anteriorly as possible, ensuring its position covers the entire area to be ablated. Use a 2.45 GHz microwave generator (AMICA-GEN, HS Hospital Service, Aprilia, Italy) to perform the ablation (20 W during 2 minutes), making sure that necrosis zone is calculated accurately to avoid damage to surrounding tissue.

**6. Kirschner Wire and Drill Insertion (**  **Fig. 2E,F****)** 

a. Kirschner Wire Placement

● Needle Removal: After verifying correct needle placement, remove the vertebroplasty needle’s inner cannula.

● Kirschner Wire Insertion: Insert a Kirschner wire into the vertebral body under fluoroscopic guidance, advancing it to the anterior third of the vertebral body (Red arrows in Fig. 2E).

● Check the correct orientation of the Kirschner wires on the axial plane using a CT scan to rule out any misalignment or to assess if and how the creation of the subsequent channels can be improved.

b. Drill Preparation

● Internal Incision: After placing the Kirschner wire, make a deep internal incision to allow the drill to advance smoothly.

● Drill Advancement: Using a working cannula and a Stryker® drill (yellow arrow in Fig. 2F), advance the drill under fluoroscopic guidance until it reaches approximately 5 mm from the anterior wall of the vertebral body. Ensure the drill is aligned with the Kirschner wire for precise positioning.

● After the removal of the first drill, an acrylic plug (pink arrow in Fig. 2F) is left in place until both bone canals have been prepared for implantation. When the drill was then removed, the working cannula was left on site to allow the subsequent introduction of the implants.

● Additionally, when the drills were withdrawn, the cavities were cleaned using Kirschner wires, obtaining bone biopsy material. Subsequent evaluation confirmed metastasis from pulmonary adenocarcinoma.

**7. SpineJack® Implantation Procedure (**  **Fig. 2G****)** 

a. Implant Preparation

● SpineJack® Kits: Prepare the expansion kit containing expandable titanium SpineJack® implants (Stryker Corp) (brown arrows in Fig. 2G), used to restore vertebral height and reduce kyphosis.

b. Simultaneous Implant Insertion

● Insertion Process: Insert two SpineJack® implants into the vertebral body through the working cannulae, one on each side.

● Under fluoroscopic guidance, deploy both implants gradually and simultaneously by turning the expander handles clockwise to restore height and reduce kyphosis.

● Total Rotation: Rotate the handles 13 full turns to fully expand the implants.

● Check the correct orientation of the SpineJack® implants on the sagittal using a CT scan to be sure of the correct position prior to the cementation.

● Final Detachment: Detach the implants by unscrewing the quick-release pin at the tip of the handles.

**8. Cement Injection** 

a. Cement Preparation

● Prepare poly-methyl-methacrylate (PMMA) bone cement (SpinePlex® radiopaque bone cement), which provides the necessary mechanical strength for stabilization.

b. Cement Injection Process

● Use a cannula to inject cement through the vertebroplasty needle.

● Fluoroscopic Monitoring: Slowly inject the cement while monitoring under real-time fluoroscopy to prevent overfilling or leakage. The cement should distribute evenly around the implants.

Final Cleaning: Clean the cannula after cement injection to prevent buildup.

**9. Final Steps and Post-procedural Assessment** 

● Cannula Removal: Gradually retract the working cannulae, ensuring no instruments are left in the vertebral body.

● CT scan: Perform a post-procedural non-contrast CT scan to assess the final placement of the implants, the symmetry of vertebral body expansion, and cement distribution. This scan also helps identify potential complications like cement leakage (Fig. 2H).

**10. Post-operative Care** 

● Pain Management: Post-procedure, discontinue NSAIDs and opioids within one week unless symptoms persist. Routine follow-up CT scans should be performed at 3 and 6 months for monitoring.

## Discussion

In this report, we present a didactic case of vertebral metastasis treated with a combined approach of microwave thermal ablation and the SpineJack system, using CT guidance in conjunction with C-arm fluoroscopy.

Our aim is to provide a detailed and technical explanation of how to perform microwave ablation in combination with the SpineJack system, utilizing both C-arm and CT guidance. This approach significantly expands the indication for the use of SpineJack in patients with pathological fractures, offering a precise and effective solution for stabilizing the spine and treating lesions in oncologic patients. By integrating these advanced imaging techniques, the procedure ensures optimal placement of the SpineJack implant while allowing for targeted tumor ablation, thus improving clinical outcomes and expanding the range of treatable cases.

This is the first detailed explanation of such an integration.

Bone metastases often cause severe pain, skeletal instability, and neurological complications, significantly reducing the quality of life for cancer patients. Standard treatments, such as chemotherapy, radiotherapy, and bisphosphonates, may be insufficient or unsuitable due to their limitations [1, 2]. Minimally invasive approaches, such as microwave thermal ablation, radiofrequency ablation, alcohol injection, interstitial laser therapy, and cryoablation, offer valuable alternatives, particularly for localized disease control [3–6].

In 2022, Ryan et al., in the CIRSE standard of practice, highlighted that vertebral augmentation techniques, including vertebroplasty, kyphoplasty, and vertebral implants, are recommended for managing vertebral compression fractures, in combination with ablative technique in selected cases [7].

Vertebral augmentation techniques, including vertebroplasty, kyphoplasty, and vertebral implants, provide spinal stabilization, especially when combined with ablative treatments to manage fractures or high-risk vertebrae.

While vertebroplasty is effective in reducing pain caused by vertebral collapse, it does not restore the height of the vertebral body or prevent spinal deformities, which can sometimes lead to additional vertebral fractures.

Additionally, vertebroplasty is not ideal for localized tumor treatment and carries a moderate risk of cement leakage. To enhance cement placement and provide better stabilization in cancer-related fractures, balloon kyphoplasty and vertebral implants have been introduced as alternatives.

The SpineJack system, with its expandable titanium implants, allows for controlled vertebral stabilization, height restoration, and partial reconstruction, all while using less cement. This results in reduced pressure on adjacent vertebrae and offers effective spinal stabilization.

As highlighted in recent literature, the SpineJack system has been associated with a lower incidence of cement leakage and a reduced risk of refracture compared to traditional techniques [8–10]. Additionally, by extracting bone material from the drill cavity of the SpineJack system, a proper bone biopsy can be performed during the same procedure. Originally designed for the treatment of post-traumatic vertebral compression fractures, the SpineJack system is now being explored for use in metastatic bone lesions and myeloma with interesting preliminary results [11–13]. In the present case, the use of Spinejack implants was combined with microwave thermal ablation, a technique that uses high-frequency electromagnetic waves to heat and destroy tumor cells, achieving effective local control, as already described in 28 patients in 2023.

CT imaging plays a critical role by providing detailed visualization of lytic lesions and allowing precise measurement of the tumor volume to be ablated. This ensures that the treatment is both effective and safe, enabling careful planning and execution of the ablation. CT guidance is essential for the accurate placement of multiple needles, especially when an extravertebral tumor component is present and needs to be treated within the same procedure.

Additionally, CT imaging aids in the positioning of protective measures for surrounding structures, such as active and passive thermal protection and electrophysiologic monitoring, which are crucial for safeguarding neural structures during the ablation.

C-arm fluoroscopy is also used in conjunction with CT to ensure the proper lateral placement of the SpineJack system. This imaging technique provides real-time monitoring of the implants’ safe and accurate expansion, as well as the controlled cementation process. The real-time visualization offered by the C-arm ensures that the cement is evenly distributed, allowing for a safer procedure with minimized risks of complications.

In this technical note, we have focused on the use of a C-arm placed laterally in the operating room, as this setup is more suitable for hospitals with limited access to advanced technologies, such as Cone Beam CT angiography systems, which can perform both CT and fluoroscopic acquisitions simultaneously. While these advanced systems offer significant advantages, this explanation is aimed at global hospital settings that may not have access to such sophisticated technology.
